# Contraception for Women with Polycystic Ovary Syndrome: Dealing with a Complex Condition

**DOI:** 10.1055/s-0042-1748036

**Published:** 2022-05-27

**Authors:** Poli Mara Spritzer

**Affiliations:** 1 Gynecological Endocrinology Unit, Division of Endocrinology, Hospital de Clínicas de Porto Alegre, Porto Alegre, RS, Brazil; 2 Department of Physiology, Universidade Federal do Rio Grande do Sul, Porto Alegre, RS, Brazil

Polycystic ovary syndrome (PCOS) is a complex condition, affecting around 9 to 13% of women at reproductive age and characterized by menstrual irregularity, ovulatory dysfunction, hyperandrogenism and polycystic ovarian morphology. Women with PCOS also present higher prevalence of obesity, cardiometabolic disturbances, such as dyslipidemia and hypertension and greater risk of impaired glucose tolerance and diabetes. Current evidence suggests that complex interactions between genetic, epigenetic, environmental, and behavioral factors contribute to the onset and to the heterogeneous clinical presentation of PCOS. In this context, and in the absence of pregnancy plans, it is essential to determine the ideal contraceptive method to offer according to the clinical, hormonal and metabolic profile of each woman. 1

Menstrual disturbances are a very common clinical feature in women with PCOS. Effective treatment will protect the endometrium from estrogen stimulation and will significantly reduce the risk of endometrial hyperplasia and cancer. Also common are signs of clinical hyperandrogenism, namely hirsutism, acne, and hair loss, which should be considered when choosing a contraceptive method. This choice may, therefore, have advantages that are not related to contraception. As part of a holistic approach to women with PCOS, even if lifestyle changes (and weight loss for overweight and obese patients) can improve or prevent metabolic disorders, their cardiometabolic profile should also be taken into account when choosing a contraceptive method.

Combined oral contraceptive (COC) is the first-line pharmacological treatment for the management of menstrual irregularities in PCOS, offering, in addition to contraception, protection of the endometrium. In addition, COC is also recommended for treating clinical hyperandrogenism, combined or not with cosmetic treatments and/or antiandrogen drugs. COC acts by suppressing LH secretion, which induces a reduction in the ovarian production of androgens. These contraceptives also increase hepatic secretion of SHBG, thereby reducing circulating free testosterone levels. 2

Current data do not support recommending specific estrogen-progestin combinations or types and doses of progestins that may be more effective than others in PCOS. 3 4 The effectiveness of COC depends, among other factors, on the duration of use, the severity of the clinical presentation, the adherence of patients to treatment. In this sense, COC containing a progestin with low affinity for the androgen receptor, such as those of the third generation (gestodene, desogestrel, norgestimate), and or a progestin with anti-androgen action (cyproterone acetate and drospirenone), could be preferred in the presence of signs of hyperandrogenism. However, the differences in efficacy between these molecules have not been clearly established. 3 In contrast, the lowest effective estrogen doses, such as 20–30 micrograms of ethinyl estradiol (EE) (or estradiol equivalent), should be considered, balancing efficacy and metabolic risk profile. 3 While the efficacy of different COC may be comparable, the risk of venous thromboembolic events should be considered before their prescription. Current data indicate that COC containing a second-generation progestin (levonorgestrel) or norgestimate have the lowest relative risk compared with other progestins. 5 6

Among women with PCOS, using COC seems to have no deleterious effect on carbohydrate metabolism, 7 but long-term, good-quality longitudinal studies are needed to confirm these data. For some overweight women with PCOS and at-risk metabolic profile or risk factors for diabetes, the combination of the COC with metformin may be considered. Lifestyle changes, including a healthy diet and regular physical activity, are then essential. 3 8 9

Although the benefits of COC in the long-term treatment of PCOS overall outweigh the risks of their use, the World Health Organization (WHO) 10 eligibility criteria for prescribing PCOS should be met. Screening for contraindications to COC is therefore imperative before any prescription in women with PCOS. 10 Indeed, in case of contraindication or refusal of COC, oral progestin-only contraceptives and long-acting, non-oral contraceptives (LARC) are good alternatives for providing endometrial protection and guarantee contraception to women with PCOS. Progestin-containing LARC includes the intrauterine device locally delivering low doses of levonorgestrel, and the subcutaneous implant, which delivers etonogestrel. Injectable medroxyprogesterone acetate is also available, but the risks outweigh the benefits of this molecule in women with PCOS who have multiple cardiovascular risk factors, such as but not limited to, high blood pressure, diabetes with chronic complications. 10 After an initial period of use, progestin-only contraceptives can cause varying degrees of endometrial atrophy and amenorrhea. But, they can also be responsible for spotting or breakthrough bleeding. 11 The choice between progestin-only contraceptives or LARC needs to be individualized, as is the case for women without PCOS, respecting the patient's preference.

Although endometrial protection is assured with oral progestin-only contraceptives and LARC, this type of contraception has no effect on hyperandrogenism in women with PCOS, with the possible exception of a recently released oral drospirenone-only oral contraceptive. 12 If the choice is for using a LARC, and in the presence of hirsutism, it is possible to consider associating an anti-androgen with the contraceptive treatment. In women with PCOS who are overweight and have cardiometabolic risk factors, including insulin resistance or dysglicemia, metformin may be also added to the contraceptive. 3 8

Regarding the copper intrauterine device, it is an effective contraceptive option and suitable for women who cannot or do not want hormonal contraception. In turn, this contraception has no effect on hyperandrogenism. Individualization of treatment is therefore recommended, combining, if necessary, an anti-androgen and/or metformin. 3

Therefore, considering the multifaceted pattern of this clinical condition, the choice of contraception in women with PCOS should be tailored to the individual needs of each patient, which may additionally include lifestyle changes and specific treatment of comorbidities such as anti-obesity, anti-hypertensive, anti-diabetic or anti-lipid drugs, when applicable.
